# Carney Complex and Its Association With Thyroid Cancer, Molecular Pathway, and Treatment

**DOI:** 10.7759/cureus.48503

**Published:** 2023-11-08

**Authors:** Chetna Sachdev, Rajesh G Gattani, Jayesh Agrawal

**Affiliations:** 1 General Surgery, Jawaharlal Nehru Medical College, Datta Meghe Institute of Higher Education and Research, Wardha, IND

**Keywords:** protein kinase a, cyclic adenosine monophosphate, carney complex, prkar1a, thyroid carcinoma

## Abstract

Thyroid cancer, being the prevailing form of endocrine malignancy, exhibits a notable surge in its incidence rates. Follicular thyroid carcinoma (FTC) and papillary thyroid carcinoma (PTC) represent the predominant well-differentiated subtypes and are recognized as the most prevalent forms of thyroid carcinomas. Over the course of several years, numerous molecular, genetic, and epigenetic modifications have been discerned in diverse forms of thyroid neoplasms. Common occurrences comprise point mutations in the *BRAF* and *RAS* genes, along with chromosomal rearrangements involving the *RET/PTC* and *PAX8/PPARγ* genes. Thyroid carcinoma, encompassing both FTC and PTC, has been documented in individuals diagnosed with Carney complex (CNC), a hereditary syndrome passed down in an autosomal dominant manner causing increased susceptibility to diverse neoplasms. CNC manifests as a result of inactivating mutations occurring within the tumor-suppressor gene known as *PRKAR1A*, which is responsible for encoding the regulatory subunit of protein kinase A (PKA) type 1α. Studies have shown that this mutation leads to activation of PKA, which, in turn, can induce FTC. In this comprehensive review, we aim to elucidate the intricate molecular mechanisms underlying thyroid tumorigenesis, specifically focusing on the deleterious consequences resulting from the deactivation of the *PRKAR1A* gene.

## Introduction and background

Carney complex (CNC) is a rare genetic disorder that follows an autosomal dominant inheritance pattern. It is distinguished by atypical pigmentation of the skin and mucous membranes, the presence of myxomas in the heart, skin, and breast, the development of endocrine tumors, the occurrence of osteochondromyxoma, psammomatous melanotic schwannoma (PMS), the formation of breast ductal adenomas, and the development of various nonendocrine tumors. Primary pigmented nodular adrenocortical disease (PPNAD), ovarian lesions, thyroid tumors, testicular tumors, and pituitary adenoma, are all instances of neoplasms in CNC affecting the endocrine system [1]. Dr. J. Aidan Carney was the first to describe the "complex of myxomas, spotty pigmentation, and endocrine overactivity" in 1985, and in 1986, it was given the name CNC [2]. CNC has been categorized as *CNC1* and *CNC2* based on the chromosome and gene involved. The development of *CNC1* is caused by germline deactivating mutations in the tumor-suppressor gene *PRKAR1A*, encoding the regulatory subunit of the cyclic adenosine monophosphate (cAMP)-dependent protein kinase A (PKA). This gene is situated on the long arm of chromosome 17 at the 17q24.2-24.3 locus. This mutation is found in 70% of familial instances of CNC [3]. The remaining cases are that of *CNC2*, where the defect is with chromosome 2p16 [4].

Thyroid cancer stands as the prevailing endocrine neoplasm within the general population, with its incidence steadily increasing in the United States [5]. This could be because of several imaging technologies such as computed tomography (CT), ultrasound, and radionuclide uptake scans that help in detecting small thyroid lumps often found by accident [6]. Approximately 90% of patients exhibit well-differentiated epithelial thyroid carcinoma, which can be further categorized based on histological criteria as either papillary or follicular thyroid cancer [7]. Follicular thyroid carcinoma (FTC) behaves more aggressively by invading the blood vessels and undergoing distant metastases [8] thus having a poorer prognosis than papillary thyroid carcinoma (PTC). Approximately 2-3% of thyroid carcinomas are medullary thyroid carcinomas (MTCs), which develop from parafollicular C cells that produce calcitonin; the remaining 7-8% of thyroid carcinomas are comprised of anaplastic carcinomas and other poorly differentiated carcinomas [7].

Thyroid malignancies are quite often noted to be associated with CNC. This is because the expansion and proliferation of thyroid gland is largely dependent on thyroid-stimulating hormone (TSH), cAMP, and PKA signaling pathway. Studies have demonstrated that the activation of PKA, facilitated by the removal of its regulatory subunit because of mutations in *PRKAR1A* tumor suppressor gene, which is responsible for *CNC1*, can induce FTC. In this review, we have extensively discussed the molecular mechanism, diagnosis, and treatment of thyroid cancer associated with CNC.

## Review

Methodology

**Figure 1:**
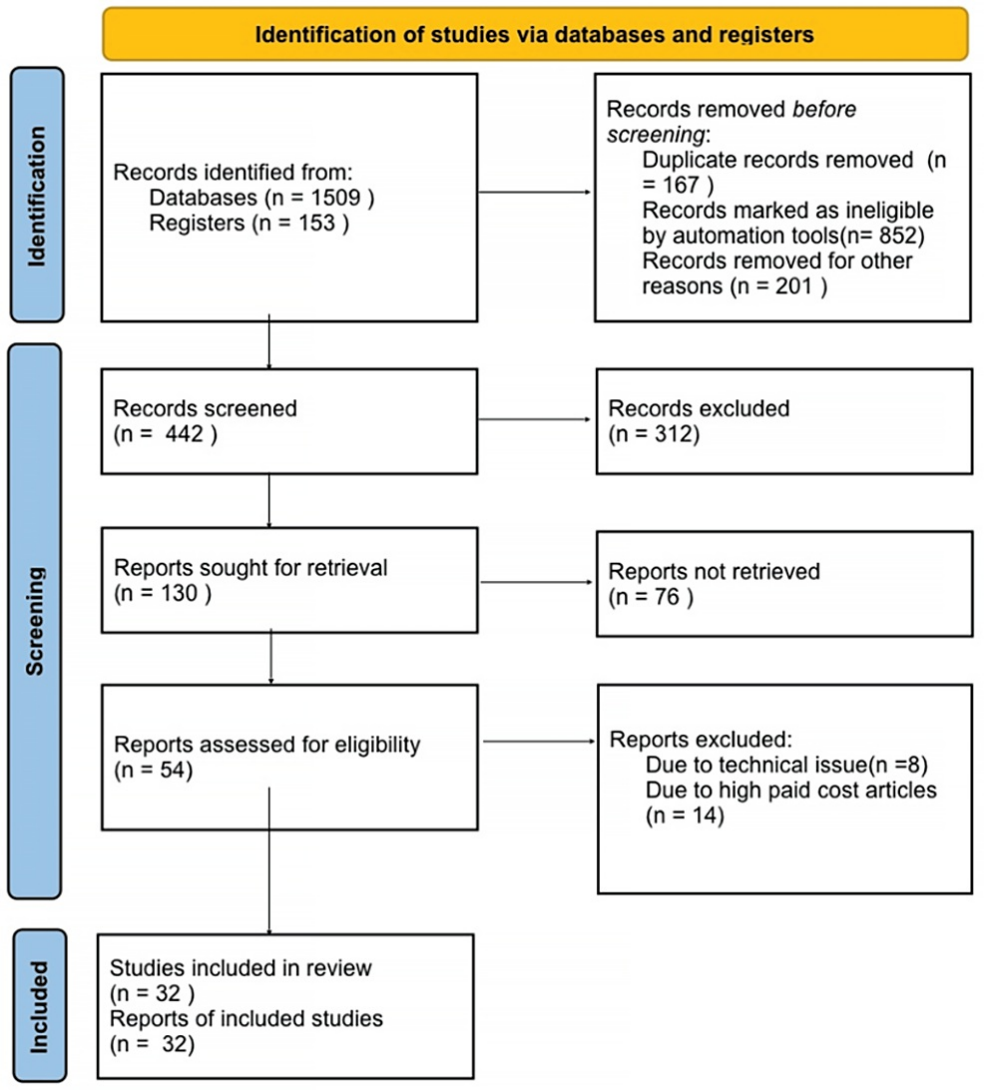
Search strategy used for this review.

PubMed was used to investigate the relevant terms and combinations: “thyroid carcinoma,” “PRKAR1A,” "carney complex,” “thyroid cancer” AND “carney complex,” “PRKAR1A mutation” AND “protein kinase A.” In this instance, only results in the English language were displayed. When multiple reports of a single study were found in the literature, the most recent reports were selected. Only review articles with novel information were considered. Figure 1 depicts the employed search strategy.

CNC and its diagnostic criteria

CNC is a rare genetic disorder, which is inherited in an autosomal dominant pattern. It is characterized by atypical pigmentation of the skin and mucous membranes, the presence of myxomas in the heart, skin, and breast, the development of endocrine tumors, the occurrence of osteochondromyxoma, PMS, the formation of breast ductal adenomas, and the development of various nonendocrine tumors. Ovarian lesions, thyroid tumors, testicular tumors, and pituitary adenoma are all instances of neoplasms in CNC affecting the endocrine system The diagnosis of CNC is established when two or more major signs of the condition are observed or when a major and a supplementary criterion is present [1]. The diagnostic criteria for CNC are mentioned in Table 1.

**Table 1:** Diagnostic criteria for CNC CNC, Carney complex The table has been created by the authors.

Criteria for the diagnosis of CNC
Major criteria
1. Spotty pigmentation of the skin and blue nevus
2. Myxoma (cutaneous, mucosal, and breast)
3. Cardiac myxoma
4. Cushing syndrome (primary pigmented nodular adrenocortical disease)
5. Growth hormone-producing pituitary adenoma causing acromegaly
6. Large cell calcifying Sertoli cell tumor
7. Psammomatous melanotic schwannomas
8. Ductal adenoma of the breast
9. Osteochondromyxoma
Supplementary criteria
1. Affected first-degree relative
2. Inactivating mutation of the *PRKAR1A* gene

Involvement of thyroid cancer in CNC

In the year 1997, Stratakis et al. conducted a study wherein they documented the presence of tumors in the thyroid gland among a cohort of 53 patients diagnosed with CNC. The prevalence of thyroid gland tumors was found to be 6%, with three individuals exhibiting such neoplasms. Specifically, one patient presented with papillary carcinoma, another with follicular carcinoma, and the third patient with follicular adenoma [9]. In a study conducted by Bertherat et al., it was observed that among a cohort of 353 patients diagnosed with CNC, a thyroid disorder was identified in 88 individuals, representing a prevalence rate of 25%. Furthermore, the occurrence of either papillary or follicular carcinoma, or a combination of both, was noted in nine out of the total 353 patients [10]. The study's findings have elucidated that the occurrence of thyroid tumors is indeed a constituent aspect of the syndrome under investigation [11].

Involvement of the thyroid gland in CNC can either be symptom-free or be linked to an exacerbated state of hyperthyroidism [12]. Familial non-medullary thyroid carcinomas (FNMTC) represent 4-8% of all primary thyroid cancers [13]. Out of these, 95% of FNMTCs are non-syndromic (alterations in FOXE1 genes), which have a similar presentation to sporadic cases when gene testing is not conducted [14]. The remaining 5% of individuals with FNMTC have germ-line mutations similar to CNC. Various other associated genetic syndromes include Cowden syndrome, Gardner syndrome, or DICER1 syndrome [15]. Follicular and nodular hyperplasia, follicular adenoma, cystic changes, PTC, and FTC are the predominant types of thyroid tumors commonly associated with CNC [16].

The age for detection of CNC is around puberty as it is a developmental disorder that is present since birth, but the diagnosis is made when the symptoms start to manifest. The thyroid nodule that appears as microcalcifications and minute can be seen as numerous hypoechoic lesions on ultrasonography around 10 years of age [1].

Molecular pathway behind thyroid cancer associated with CNC

CNC is a disorder that exhibits both clinical and molecular diversity. As previously indicated, the application of genetic linkage analysis has yielded noteworthy findings regarding CNC. Specifically, two separate loci have been identified, namely, the *CNC1* locus located on chromosome 17q22-24, encompassing the *PRKAR1A* gene responsible for encoding the R1α subunit of PKA, and the *CNC2* locus located on chromosome 2p16 [17]. PKA is a highly prevalent serine-threonine kinase that operates in a cAMP-dependent manner. It has a critical role in governing various cellular mechanisms such as metabolism, transcription, cell cycle advancement, and programmed cell death. PKA is formed by the assembly of four subunits, two regulatory and two catalytic, forming a heterotetramer. The regulatory subunits and catalytic subunits of PKA are known to exhibit four isoforms each (R1α, R2α, R1β, and R2β of regulatory and Cα, Cβ, Cγ, and PRKX of catalytic). These isoforms possess distinct localization patterns and specificity profiles, thereby contributing to the functional diversity of PKA [18].

**Figure 2:**
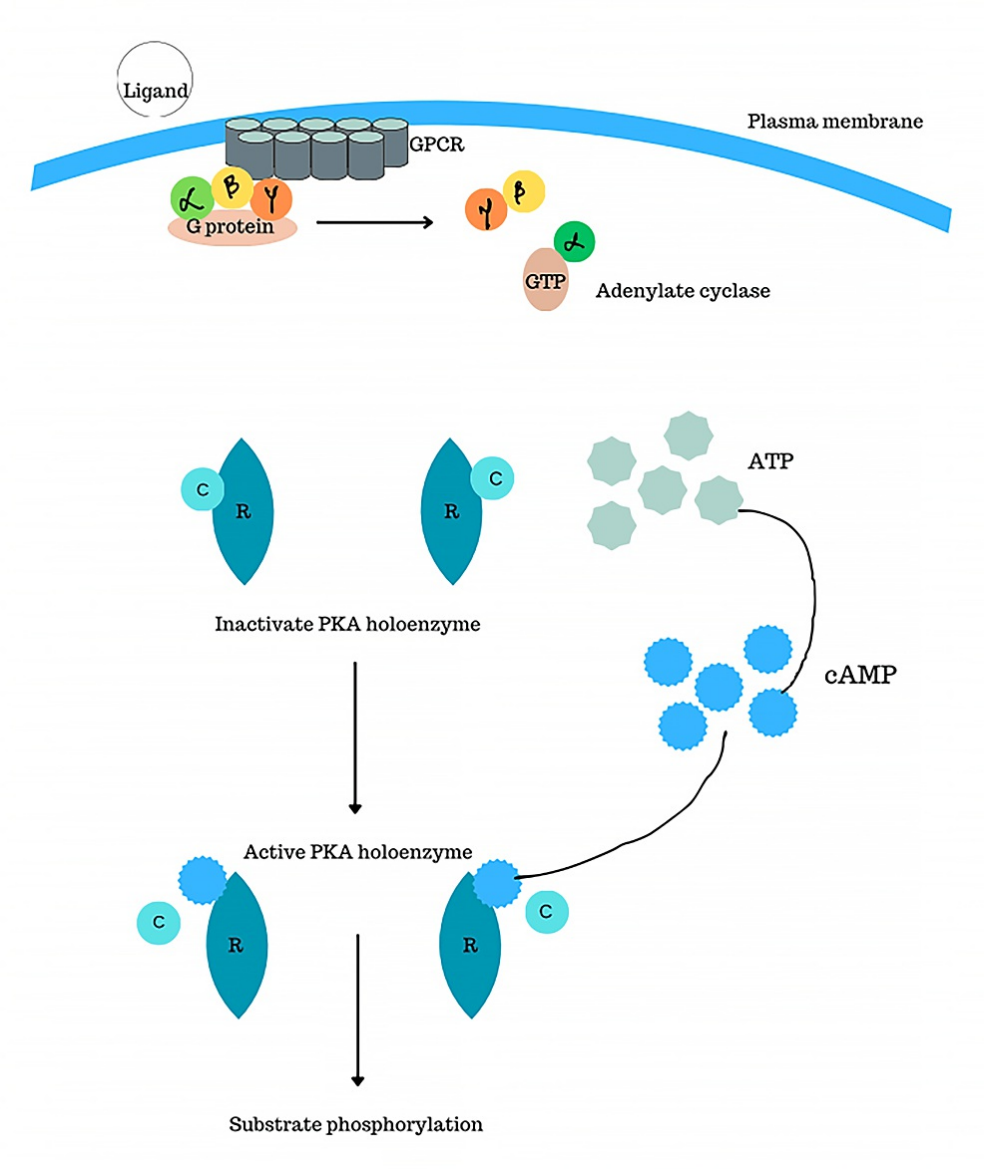
Molecular pathway involved in the PRKAR1A mutation ATP, adenosine triphosphate; C, catalytic subunit; cAMP, cyclic adenosine monophosphate; GPCR, G protein-coupled receptors; GTP, guanosine triphosphate; PKA, protein kinase A; R, regulatory subunit Figure has been created by the author.

The typical mechanism behind the formation of endocrine cancers in CNC involves binding of various ligands related to the endocrine system, such as adrenocorticotropic hormone, growth hormone releasing hormone, follicle-stimulating hormone, melanocytes-stimulating hormone, and TSH, to the G-protein coupled receptor, resulting in the activation of PKA under physiological conditions. The *PRKAR1A* gene is responsible for encoding the R1α subunit of PKA [19]. Following the interaction between cAMP and the regulatory (R) subunits, a notable change in their conformation occurs, leading to the subsequent separation of every active catalytic (C) subunit from the dimeric complex formed with its respective R subunit. Following this, liberated C subunits stimulate the addition of phosphate groups to serine and threonine amino acid residues within proteins, which play a crucial role in activating subsequent processes [20]. The activation of PKA within the thyroid gland by the production of cAMP signals influences both cell differentiation and cell proliferation. Elevated TSH levels in humans have been observed to stimulate tumorigenesis [21]. Hence, the *PRKAR1A* mutation exerts a direct impact on the thyrocytes and also on other endocrine cells such as adrenocortical cells, somatotrophs, melanocytes, and Sertoli cells, leading to other associated endocrine neoplasms. The mechanism is shown in Figure 2.

CNC does not exhibit involvement of the conventional thyroid cancer pathways, namely, the MAPK and PIK3-AKT pathways [22]. The latest in vitro research indicates that PKA stimulates AMP-activated kinase (AMPK) in Carney-related FTC via serine/threonine kinase 11 without inhibiting the mammalian target of rapamycin (mTOR) activation [23].

Screening and surveillance

Each individual with confirmed or suspected CNC should receive comprehensive care from a multi-disciplinary team tailored to address the specific manifestations of the disease. Additionally, they should be provided with regular follow-up and genetic counseling services to ensure long-term management and support. Patients with CNC can be diagnosed with clinical and biochemical screenings, whereas testing for *PRKAR1A* mutation is not necessary unless there is a family history of mutation. DNA testing is considered the most efficacious method for screening individuals who are suspected of CNC. However, the accuracy of DNA testing is very limited [4]. This constraint implies that molecular diagnosis is unlikely to take over clinical diagnosis in the foreseeable future.

Given that CNC exhibits autosomal dominant inheritance, it follows that the offspring of an afflicted individual possess a 50% probability of receiving the disease-causing allele. Most of the people with the disease (around 70%) have a parent with the disease. The harmful gene is known to exist in the family, and hence prenatal testing must be the first recommendation. The significance of exploring thyroid nodules in individuals with CNC has been demonstrated by human studies. Thyroid ultrasound reveals the existence of multiple thyroid nodules in a substantial proportion, approximately 75%, of the patients diagnosed with CNC. Most of these nodules are non-functioning follicular adenomas; however, thyroid carcinomas are also prevalent. The timely identification of cancer is of utmost importance, and individuals diagnosed with CNC should undergo ongoing clinical or ultrasound monitoring, along with a biopsy of any nodules that raise suspicion, to facilitate the early discovery of carcinomas. The observed rise in family cases can be attributed to the implementation of a stringent screening decorum for all first-degree relatives of affected individuals [24,25].

The clinical surveillance protocol for patients diagnosed with CNC varies depending on the age group of the individuals. It is recommended that adolescent and adult patients undergo a yearly evaluation consisting of various diagnostic procedures. These procedures include an echocardiogram, testicular and thyroid ultrasound, and measurement of urinary free cortisol (UFC) and serum insulin-like growth factor 1 (IGF-1) levels [12]. These assessments aim to provide valuable insights into the cardiovascular health, reproductive organs, and thyroid gland, as well as hormonal balance in individuals within this specific age group. In cases where meticulous observation of pubertal staging and growth rate reveals potential irregularities, such as suspected Cushing syndrome, it is imperative to conduct the necessary diagnostic evaluations. In the context of PPNAD-induced Cushing syndrome, it is advised to incorporate diurnal cortisol level assessments at specific time points (11.30 pm, 12.00 MN, 7.30 am, and 8.00 am) alongside UFC measurements. Additionally, the utilization of a dexamethasone-stimulation test and adrenal CT are recommended diagnostic procedures. In the evaluation of gigantism/acromegaly, it is recommended to consider the assessment of serum IGF-1 levels, along with the utilization of pituitary magnetic resonance imaging (MRI) and a 3-hour oral glucose tolerance test as diagnostic tools. PMS, a rare neoplasm originating from Schwann cells, often requires comprehensive evaluation using MRI techniques. In order to accurately evaluate the extent and potential metastasis of this tumor, MRI scans of the brain, abdomen, spine, retroperitoneum, chest, or pelvis may be deemed essential [4].

Treatment

In the field of CNC, it is imperative to approach each individual complication or tumor as a distinct entity, warranting separate attention and consideration. Surgically removing cardiac myxomas is the recommended course of action; however, a significant number of patients undergo multiple open-heart surgeries due to the recurrence of tumors. The medical management of growth hormone producing pituitary adenomas typically involves the administration of somatostatin analogs or surgical intervention for tumor removal. In the context of PPNAD, the optimal therapeutic approach entails surgical intervention through bilateral adrenalectomy. Nevertheless, in specific instances, medical adrenalectomy utilizing steroidogenesis inhibitors such as ketoconazole or mitotane may be considered as an alternate option [26]. Cutaneous and breast myxomas may need to be surgically excised, but since these are benign conditions, it is not always required. Fine-needle aspiration of thyroid lumps is suggested in cases that look suspicious, and thyroid cancer can be treated based on its histology [1].

Treatment of thyroid cancer associated with CNC

The best opportunity for achieving a cure in cases of familial MTC is by the complete surgical removal of the tumor prior to its change into a malignant state or its spread beyond the thyroid gland. Children who possess MEN2B (multiple endocrine neoplasia 2A) or RET codon mutations exhibit the most severe manifestations of MTC. As such, it is recommended that these children receive total thyroidectomy before reaching six months of age [27]. To date, the administration of systemic chemotherapy in individuals diagnosed with locally advanced or metastatic MTC has had limited clinical responses. The therapeutic approach of angiogenesis, with a particular focus on vascular endothelial growth factor (VEGF) receptors, has yielded the most notable clinical outcomes thus far in the treatment of MTC [28].

Nonetheless, the eventual advancement of disease while undergoing therapy with VEGF receptor inhibitors implies that the activation of alternative pathways facilitates the proliferation of tumors and their metastasis to other body areas [29].

Both cabozantinib and vandetanib have been granted approval for the therapeutic management of MTC. Vandetanib has been granted approval for the management of symptomatic, unresectable, locally advanced, or metastatic MTC in patients, as determined by the findings of a phase 3 clinical trial [30]. FDA has granted approval for the use of lenvatinib and sorafenib, both classified as multikinase inhibitors (MKIs), in the management of advanced differentiated thyroid cancer. The approval of sorafenib was granted on the basis of the positive outcomes observed in a phase 3 clinical trial that employed a placebo-controlled design. Currently, immunotherapy trials are also going on for the management of thyroid cancers. Pembrolizumab (programmed cell death protein - 1, i.e., PD-1 checkpoint inhibitor) in trials displayed to decrease size of PTC and FTC by 35-50%. Spartalizumab is another PD-1 inhibitor effective against anaplastic thyroid carcinoma [31,32].

However, currently, there is a lack of medicinal interventions specifically designed to modulate the cAMP/PKA signaling pathway in individuals with CNC. Hence, the preferred therapeutic approach for patients with *PRKAR1A*-associated thyroid tumor is surgical intervention [33]. The findings of different studies included in this article are shown in Table 2.

**Table 2:** Findings of the different studies included in this review.

Author name	Year	Country of origin	Findings
Espiard et al. [1]	2013	France	Provides an overview of Carney complex, a genetic disorder associated with multiple neoplasia and lentiginosis.
Amieux et al. [2]	2002	USA	Highlights the crucial role of RI alpha in maintaining regulated PKA activity.
Kirschner et al. [3]	2000	USA	Identifies mutations in the *PRKAR1A* gene associated with Carney complex.
Stratakis et al. [4]	2011	USA	Discusses Carney complex as a familial syndrome linked to chromosome 2.
Pringle et al. [5]	2014	USA	Demonstrates the dual activation of PKA and mTOR in follicular thyroid cancers.
Raman, et al. [6]	2014	USA	Discusses the Pax-8-PPAR-γ fusion protein in thyroid carcinoma.
Zhang et al. [7]	2015	USA	Examines genomic binding and regulation of gene expression by the PAX8-PPARG fusion protein.
Antico-Arciuch et al. [8]	2010	USA	Explores cross-talk between PI3K and estrogen in thyroid carcinogenesis.
Stratakis et al. [9]	2011	USA	Describes thyroid gland abnormalities in patients with Carney complex.
Bertherat et al. [10]	2009	France	Analyzes mutations in the *PRKAR1A* gene and their phenotypic effects in Carney complex.
Nagy et al. [11]	2011	USA	Reports the frequency of germline PTEN mutations in differentiated thyroid cancer.
Carney et al. [12]	2018	USA	Discusses the spectrum of thyroid gland pathology in Carney complex, emphasizing the importance of follicular carcinoma.
Peiling Yang and Ngeow [13]	2016	Singapore	Explores the genetic factors contributing to familial non-medullary thyroid cancer.
Hińcza et al. [14]	2019	Poland	Provides an overview of current knowledge on germline genetic risk factors for non-medullary thyroid cancer.
Cirello [15]	2021	Italy	Discusses clinicopathological features and genetic risk factors in familial non-medullary thyroid carcinoma.
Kamilaris et al. [16]	2019	USA	Provides an overview of Carney complex, highlighting clinical features and genetic aspects.
Matyakhina et al. [17]	2003	USA	Identifies chromosome 2 abnormalities in Carney complex tumors.
Taylor et al. [18]	2012	USA	Discusses the assembly of allosteric macromolecular switches, drawing lessons from PKA.
Griffin et al. [19]	2004	USA	Demonstrates that down-regulation of *PRKAR1A* leads to endocrine and other tumors.
Griffioen et al. [20]	2002	Belgium	Explores molecular mechanisms controlling the localization of protein kinase A.
Haymart et al. [21]	2008	USA	Reports a correlation between higher serum thyroid stimulating hormone levels and increased risks of thyroid cancer and advanced tumor stage.
Pringle et al. [22]	2012	USA	Demonstrates that thyroid-specific ablation of *PRKAR1A* leads to hyperthyroidism and follicular thyroid cancer.
Kari et al. [23]	2019	USA	Shows how PKA activates AMPK through LKB1 signaling in follicular thyroid cancer.
Carney [24]	2010	USA	Describes psammomatous melanotic schwannoma as a distinctive, heritable tumor with unique associations.
Sandrini et al. [25]	2003	USA	Discusses the clinical and molecular genetics of Carney complex.
Correa et al. [26]	2015	USA	Provides an update on Carney complex, highlighting clinical aspects.
Moo-Young. et al. [27]	2009	USA	Reviews sporadic and familial medullary thyroid carcinoma.
Sherman [28]	2009	USA	Discusses the effects of tyrosine kinase inhibitors on the thyroid.
Bergers and Hanahan et al. [29]	2008	USA	Explores modes of resistance to anti-angiogenic therapy.
Wells et al. [30]	2012	USA	Reports results from a phase 3 trial on vandetanib in medullary thyroid cancer.
Brose et al. [31]	2014	USA	Presents findings from a phase 3 trial on sorafenib in radioactive iodine-refractory differentiated thyroid cancer.
Cabanillas et al. [32]	2015	USA	Shares outcomes from a phase 2 trial of lenvatinib in radioiodine-refractory differentiated thyroid cancer.
Bouys and Bertherat [33]	2021	France	Provides a clinical and genetic update on Carney complex two decades after the identification of the *PRKAR1A* gene.

## Conclusions

In summary, the progress made in comprehending the molecular pathways linked to thyroid cancer has resulted in improved prognostic and diagnostic capacities for this particular ailment. *PRKAR1A*, a pivotal regulator of PKA activity, potentially plays a role in the intricate molecular mechanisms that underlie the pathogenesis of thyroid cancer associated with CNC. The identification of the genetic underpinnings of *PRKAR1A*-related thyroid malignancies and understanding the molecular mechanisms that underlie are of utmost importance, as it will enable the development and advancement of clinical care and treatment strategies that specifically target the PKA pathway and trigger thyroid cancer in patients affected with CNC.
